# Intraocular Pressure Is a Poor Predictor of Hydration Status following Intermittent Exercise in the Heat

**DOI:** 10.3389/fphys.2017.00036

**Published:** 2017-02-01

**Authors:** Ian B. Stewart, Brittany Dias, David N. Borg, Aaron J. E. Bach, Beatrix Feigl, Joseph T. Costello

**Affiliations:** ^1^School of Exercise and Nutrition Sciences and Institute of Health and Biomedical Innovation, Kelvin Grove, Queensland University of TechnologyBrisbane, QLD, Australia; ^2^School of Biomedical Sciences and Institute of Health and Biomedical Innovation, Kelvin Grove, Queensland University of TechnologyBrisbane, QLD, Australia; ^3^Extreme Environments Laboratory, Department of Sport and Exercise Science, University of PortsmouthPortsmouth, UK

**Keywords:** hydration assessment, eye, intraocular pressure, dehydration, exercise, heat

## Abstract

Current hydration assessments involve biological fluids that are either compromised in dehydrated individuals or require laboratory equipment, making timely results unfeasible. The eye has been proposed as a potential site to provide a field-based hydration measure. The present study evaluated the efficacy and sensitivity of intraocular pressure (IOP) to assess hydration status. Twelve healthy males undertook two 150 min walking trials in 40°C 20% relative humidity. One trial matched fluid intake to body mass loss (control, CON) and the other had fluid restricted (dehydrated, DEH). IOP (rebound tonometry) and hydration status (nude body mass and serum osmolality) were determined every 30 min. Body mass and serum osmolality were significantly (*p* < 0.05) different between trials at all-time points following baseline. Body mass losses reached 2.5 ± 0.2% and serum osmolality 299 ± 5 mOsmol.kg^−1^ in DEH. A significant trial by time interaction was observed for IOP (*p* = 0.042), indicating that over the duration of the trials IOP declined to a greater extent in the DEH compared with the CON trial. Compared with baseline measurements IOP was reduced during DEH (150 min: −2.7 ± 1.9 mm Hg; *p* < 0.05) but remained stable in CON (150 min: −0.3 ± 2.4 mm Hg). However, using an IOP value of 13.2 mm Hg to predict a 2% body mass loss resulted in only 57% of the data being correctly classified (sensitivity 55% and specificity 57%). The use of ΔIOP (−2.4 mm Hg) marginally improved the predictive ability with 77% of the data correctly classified (sensitivity: 55%; specificity: 81%). The present study provides evidence that the large inter-individual variability in baseline IOP and in the IOP response to progressive dehydration, prevents the use of IOP as an acute single assessment marker of hydration status.

## Introduction

Current best-practice human hydration assessments include osmolality of blood, saliva, or urine; specific gravity or color of urine; and changes in body mass compared to a baseline collected over several days (Armstrong, 2007; Cheuvront et al., 2010, 2013; Kenefick and Cheuvront, 2012). These procedures are either expensive, invasive, require clinical laboratory equipment, rely on a non-dehydrated baseline criterion or on body fluids that are compromised in a dehydrated individual. Reviews of hydration assessment techniques have highlighted the need to develop field indices that are suitable for the evaluation of large groups of people, involved in athletic or challenging occupational situations, where dynamic (involving a baseline criterion) measurements are not necessary (Armstrong, 2007).

Recently the eye has been identified (Sollanek et al., 2012; Sherwin et al., 2015) as having the potential to provide a valid hydration assessment in field settings, where the use of invasive procedures is limited. The relationship between ocular fluids (tear and aqueous humor), blood pressure and plasma osmolality has provided a case for tear fluid osmolarity (Fortes et al., 2011), tear break-up time (Sweeney et al., 2013), and intraocular pressure (IOP) (Hunt et al., 2012) as potential non-invasive measures of hydration status.

IOP is governed by the rates of formation and drainage of aqueous humor. Aqueous is continually being formed, filtering from the capillaries in the ciliary processes, flowing through the anterior chamber, and draining from the eye through the limbus and the scleral venous sinus. The production of aqueous humor is under tight neuro-endocrine regulation; with its flow through the anterior chamber influenced by hydrostatic, oncotic and osmotic pressures and its outflow regulated by the autonomic nervous system (Coca-Prados and Escribano, 2007).

Hyperosmolality of the blood caused by high intensity short duration exercise has been associated with reduced IOP (Marcus et al., 1970; Stewart et al., 1970). Several researchers have also suggested that low intensity long duration exercise in a hot environment resulting in sweating induced hypovolemia and subsequent hyperosmolality (as opposed to acidosis from high intensity exercise) could lower the rate of aqueous formation and consequently reduce IOP (Marcus et al., 1970; Harris et al., 1994). However, these studies did not require participants to exercise for a sufficient duration, or in a hot environment, to elicit a change in hydration status.

To date only two studies have assessed IOP over a prolonged duration and/or in a hot environment where an individual would experience significant body mass losses using different methods of IOP assessment. The first involved a 24 h march (17–32°C, 45–85% relative humidity) where IOP progressively declined for the first 15 h, at which time serum osmolality peaked (Ashkenazi et al., 1992). Forty-eight hours after completing the march, a reduction in IOP was observed, and again was accompanied by a rise in serum osmolality. At both time points a statistically significant moderate correlation (*r* = −0.679 and −0.649, respectively, *p* < 0.001) between IOP and serum osmolality was observed (Ashkenazi et al., 1992). More recently a small sample pilot study required participants to complete three 30 min walking bouts in a controlled environment (43°C, 20% relative humidity) (Hunt et al., 2012) and observed statistically significant moderate relationships between IOP and plasma osmolality (*r* = −0.682), and change in body mass (*r* = 0.507).

Currently, the efficacy and sensitivity of IOP to determine changes in body mass associated with sweating induced hypovolemia have only been conducted in uncontrolled environments (Ashkenazi et al., 1992) or in a small pilot study (Hunt et al., 2012). Due to the potential feasibility of using IOP as a field based measure of hydration status in various sporting, occupational and clinical settings, the aim of the present investigation was to determine if IOP was associated with hydration status (body mass loss and serum osmolality) following exercise in the heat with and without fluid restriction. It was hypothesized that IOP would be reduced to a greater extent during exercise with fluid restriction, concomitant with modest hypohydration (>2% body mass loss) and increased serum osmolality.

## Methods

### Ethical approval

The testing protocols carried out in this study were approved by the Queensland University of Technology Human Research Ethics Committee. Participants were informed of the procedures and had any questions answered to their satisfaction prior to giving their oral and written consent to participate. The study conformed to the current Declaration of Helsinki guidelines.

### Participants

Twelve healthy, physically active males (mean ± SD): age 24 ± 2 year, height 178 ± 6 cm, mass 75 ± 7 kg, $\dot{\text{V}}$O_2max_ 56 ± 4 mL·kg^−1^·min^−1^, sum of eight skinfolds 75 ± 29 mm) with normal ocular health as confirmed by an optometrist volunteered to participate. Exclusion criteria included any history of ocular disease involving raised eye pressure (or existing glaucoma or ocular hypertension).

### Experimental design

Participants were required to attend the laboratory on three occasions. The first laboratory visit involved eye testing, to determine high contrast visual acuity (Snellen chart) and health of the anterior and posterior eye (slit lamp biomicroscopy, funduscopy and IOP) by an experienced optometrist. The first visit also involved the determination of maximal aerobic power by an incremental treadmill running test to exhaustion and skin fold assessment of body composition, as previously described (Stewart et al., 2014). The remaining two trials, separated by a minimum of 7 days, involved five 30 min walking bouts. To control for the effects of circadian rhythm on IOP both walking trials commenced at the same time of day and differed only in the provision of fluid, with the participants either receiving no fluid throughout (to induce body mass losses, DEH) or fluid replacement (with the aim to maintain body mass, CON). The order of the two walking trials was counterbalanced across participants.

### Experimental protocol

The two walking trials followed a similar protocol. Participants were asked to avoid heavy exercise and the consumption of alcohol, caffeine and tobacco in the 24 h prior to each walking trial. To ensure euhydration, participants were instructed to consume 30 mL·kg^−1^ body mass of fluid (either water or sports drink) between 4 and 10 pm the night before each session, and a further 250 mL of fluid the morning of the trial (at least 1 h prior to trial commencement). The participants were also given a calibrated (Hunt and Stewart, 2008) ingestible core temperature sensor (CorTemp, HQ Inc, Palmetto, FL, USA) to swallow the evening prior.

Upon arriving at the laboratory participants were asked to collect a mid-stream urine sample that was assessed for specific gravity (USG). Participants with a USG value < 1.020 were classified as euhydrated (23 of 24 trials) and those with higher values (1 of 24 trials) were provided with an additional 500 mL of water to be consumed prior to the commencement of the walking trials. A chest strap (Polar Team2, Kempele, Finland) and data logger (CorTemp, HQ Inc, Palmetto, FL, USA) were then fitted to provide continuous heart rate and core temperature recordings, respectively.

Participants were then seated and a cannula was inserted in the left antecubital fossa to attain venous blood samples. Following at least 10 min of seated rest IOP and blood pressure from the right arm, using the auscultatory method, were obtained and blood samples drawn. Intraocular pressure was measured by an optometrist using a handheld contact (rebound) tonometer (TA01i, icare®, Helsinki, Finland). The device measures the IOP in <0.1 s and averages six readings to minimize deviation and to produce a calculated measurement value. The IOP measurement was performed in duplicate (triplicate if difference was > 1 mm Hg) for the right eye only (Fernandes et al., 2005). The closest two IOP values were used to obtain an average intraocular pressure for the participant for each time point. Blood samples were collected into 5 mL serum separating vacutainers for the determination of serum osmolality, 6 mL K3 EDTA vacutainers for the determination of hemoglobin concentration (Hb), haematocrit (Hct) and blood lactate (Stewart et al., 2005). Hb and Hct were used to calculate the percent change in plasma volume (PV) during the trial (Dill and Costill, 1974). Nude body mass measurements were then obtained to the nearest 50 g (Tanita BWB-600, Wedderburn, Australia).

Participants then entered the environmental chamber (40°C, 20% relative humidity, 4.7 km·h^−1^ air flow) and commenced walking at 5 km·h^−1^ and 1% gradient with core temperature and heart rate recorded and monitored continuously. Following 30 min the participants were removed from the environmental chamber into an air-conditioned laboratory and had 10 min of seated rest, after which IOP, blood pressure, blood collection, and nude body mass (after towel drying) were determined, in that order. This was repeated five times for a total of 150 min walking which equated to a total distance of 12.5 km for all participants.

During the fluid provision trial, 300 mL of room temperature (~22°C) water was provided in the first 30 min walking bout and in the remaining four walking bouts water provision was equated to the body mass loss in the preceding walking bout. To ensure the fluid consumption had no subsequent effect on the measurement of IOP all fluid was consumed within the first 10 min of the walking bout (Brucculeri et al., 1999). Food, two biscuits and a banana, equating to a weight of ~90 g, was provided in both trials every hour.

### Statistical analysis

A power calculation using G*Power 3 software was performed in order to determine the required sample size for the experiment. Using an effect size from data previously collected in our laboratory (Cohen's *d* = 0.8, *n* = 7; Hunt, 2011), with α and power levels set at 0.05 and 0.8, respectively, a sample of twelve participants was calculated to provide sufficient statistical power to detect changes in IOP during progressive dehydration.

The normal distribution of data was confirmed using descriptive methods (kurtosis, skewness, outliers and distribution plots) and inferential statistics (Shapiro–Wilk Test). Continuous variables were summarized as mean ± standard deviation (unless otherwise stated). A two way repeated measures analysis of variance (ANOVA) was performed to assess the effects of time (baseline, 30, 60, 90, 120, and 150 min) and trial (DEH and CON) on IOP, indicators of hydration status, heat strain, and blood pressure variables. Post-hoc analysis, using a Bonferroni correction, were conducted where appropriate. A Pearson's correlation coefficient was determined to observe the relationship between IOP and indicators of hydration status, heat strain, blood pressure and lactate across all trials and time points. Where a statistically significant relationship was observed, a univariate general linear model, with participant ID as a random effect, was utilized to determine statistical significance. This was to account for the within-participant correlation likely present within the data (due to repeated measures), and provides an average equation of the linear association from the association within each participant. Confidence intervals around the slope of the line were calculated using the t statistic for eleven degrees of freedom. Finally, the sensitivity and specificity of IOP and ΔIOP to identify a 2% loss in body mass, in accordance with the ACSM Position Stand in Exercise and Fluid Replacement (Sawka et al., 2007) and other recent literature (Muñoz et al., 2013; Cheuvront and Kenefick, 2014) was determined. Statistical significance for all analysis was set at the *p* < 0.05 level.

## Results

### Baseline data

IOP, body mass (CON 76.3 ± 8.4, DEH 76.2 ± 8.7 kg), serum osmolality, core temperature, heart rate, mean arterial pressure and blood lactate were similar (*p* > 0.05; Table 1) at baseline before each trial.

**Table 1:** **Physiological changes observed during the fluid restriction (DEH) and provision (CON) trials**.

	**Baseline**	**30 min**	**60 min**	**90 min**	**120 min**	**150 min**
**IOP (mm Hg)**						
CON	14.4 ± 4.1	15.5 ± 3.9	14.7 ± 3.9	14.1 ± 4.0	14.5 ± 3.5	14.2 ± 4.0
DEH	15.6 ± 3.5	14.2 ± 3.5	14.8 ± 4.1	13.3 ± 3.3	13.2 ± 3.6	13.0 ± 3.0
**Δ BODY MASS (%)**						
CON		0.0 ± 0.1	−0.1 ± 0.1	−0.1 ± 0.1	−0.1 ± 0.2	−0.2 ± 0.2
DEH		−0.5 ± 0.1^†^	−1.0 ± 0.1^†^	−1.5 ± 0.1^†^	−2.0 ± 0.2^†^	−2.5 ± 0.2^†^
**SERUM OSMOLALITY (mOsmol·kg^−1^)**						
CON	291 ± 5	291 ± 3	291 ± 4	291 ± 3	292 ± 4	292 ± 3
DEH	292 ± 3	293 ± 3^*^	294 ± 3^*^	297 ± 4^*^	298 ± 4^*^	299 ± 5^*^
**CORE TEMPERATURE (°C)**						
CON	37.2 ± 0.3	37.4 ± 0.2	37.5 ± 0.2	37.6 ± 0.2	37.6 ± 0.2	37.6 ± 0.2
DEH	37.1 ± 0.3	37.4 ± 0.2	37.6 ± 0.2	37.7 ± 0.2^*^	37.9 ± 0.2^*^	38.0 ± 0.2^*^
**HEART RATE (b·min^−1^)**						
CON	68 ± 7	72 ± 12	74 ± 12	78 ± 13	78 ± 12	79 ± 13
DEH	66 ± 9	74 ± 16	77 ± 16	83 ± 17	89 ± 18^*^	96 ± 19^*^
**MEAN ARTERIAL PRESSURE (mm Hg)**						
CON	89 ± 8	88 ± 8	88 ± 6	88 ± 5	88 ± 6	89 ± 6
DEH	90 ± 6	91 ± 6	91 ± 8	90 ± 7	91 ± 7	89 ± 9
**BLOOD LACTATE (mmol·L^−1^)**						
CON	1.03 ± 0.46	0.98 ± 0.44	0.73 ± 0.47	0.93 ± 0.54	0.77 ± 0.41	0.94 ± 0.56
DEH	1.31 ± 0.74	0.92 ± 0.49	0.89 ± 0.57	1.16 ± 1.06	1.09 ± 0.85	1.20 ± 0.81

Data are mean ± SD (n = 12). Significantly different to control at same time point

* (p < 0.05);

† *(p < 0.001)*.

### Dehydration protocol

All twelve participants completed the 150 min of exercise in the CON and DEH trials and no adverse events were recorded. DEH resulted in significant (*p* < 0.001) body mass losses and increases in serum osmolality compared with the CON trial (Table 1). Plasma volume was also significantly reduced in the DEH compared with the CON trial (DEH–CON: −5.1 ± 3.4%, *p* = 0.001, *n* = 10). No significant differences were observed in mean arterial pressure or blood lactate concentration, however heart rate and core temperature were significantly elevated (*p* < 0.05) in the DEH trial at the 120 and 150 min and 90, 120, and 150 min time points, respectively (Table 1).

### IOP

The typical error of measurement for IOP, utilizing the baseline data from both trials, was calculated to be 1.65 mm Hg. No significant main effect for trial was observed (CON 14.6 ± 3.7, DEH 14.0 ± 3.3 mm Hg, *p* = 0.257). A significant main effect for time (*p* < 0.001) and trial by time interaction was observed for IOP (*p* = 0.042, Table 1), indicating that over the duration of the trials IOP declined to a greater extent in the DEH compared with the CON trial. However, utilizing a Bonferroni correction for multiple comparisons, no single time-point maintained statistical significance. Similarly, when using the LSD post hoc analysis, no differences were observed.

Significant correlations (*p* < 0.05) were observed between IOP and body mass loss (*r* = 0.181), blood pressure (*r* = 0.501), and blood lactate (*r* = 0.190). As such these variables were entered into a univariate general linear model as covariates (individually) with IOP as a dependent variable and participant number as a random factor, to account for the repeated measurements. Only body mass loss was found to be significantly associated (Table 2).

**Table 2:** **Univariate general linear model for IOP, Δ IOP, and covariates, with participant ID as a random factor**.

	**F**	**Degrees of freedom**	**Significance**	**Intercept (SE)**	**Slope**	**95% CI Low**	**95% CI High**
**ABSOLUTE IOP**							
Δ Body mass ^*^	22.096	1, 107	<0.001	14.75 (1.164)	0.77	0.41	1.13
Blood pressure	0.552	1, 131	0.459	4.91 (3.92)	0.03	−0.05	0.01
Blood lactate	0.004	1, 129	0.952	14.29 (1.01)	0.02	−0.66	0.70
**Δ IOP**							
Δ Body mass ^*^	56.352	1, 107	<0.001	0.26 (0.88)	1.33	0.94	1.72
Serum osmolality	62.920	1, 106	<0.001	94 (41)	−323	−412	−233
Core temperature	22.976	1, 101	<0.001	127.78 (1.58)	−3.42	−4.99	−1.85

* *Only data at 30, 60, 90, 120, and 150 min time points was used in this analysis as baseline values were “0” for all participants*.

*SE–standard error of intercept*.

*95% CI–95% confidence interval around the slope of the line*.

**Figure 1:**
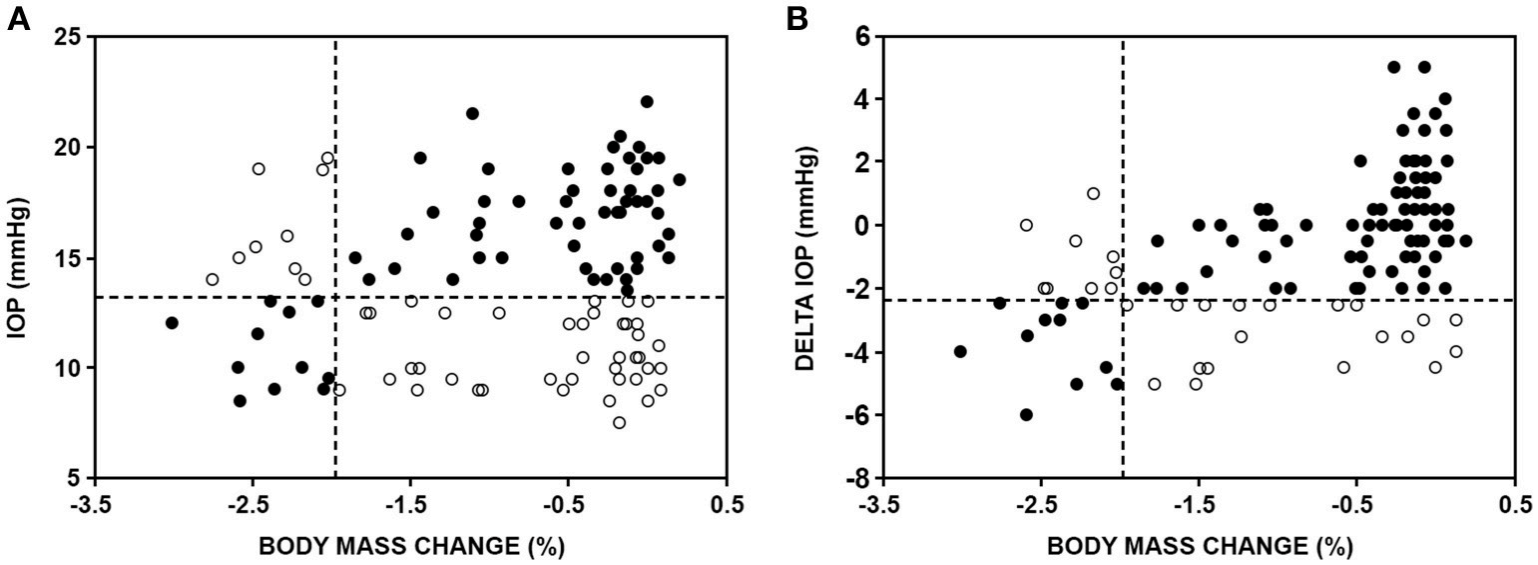
**(A)** Sensitivity and specificity capability of IOP using a 13.2 mm Hg criterion value to assess a 2% body mass loss. Dashed lines represent −2% body mass change and 13.2 mm Hg IOP. **(B)** Sensitivity and specificity capability of a ΔIOP using a −2.4 mm Hg criterion value to assess a 2% body mass loss. Dashed lines represent −2% body mass change and −2.4 mm Hg IOP. Solid circles represent correct classification (true positive and negative) and open circles incorrect classification (false positive and negative).

When a body mass loss of 2% (Sawka et al., 2007) was taken as a criterion limit for the presence of hypohydration using the regression equation, IOP was predicted to be 13.2 mm Hg. Figure 1A displays the relationship of IOP and body mass loss for each participant, with reference to these cut-off limits for hydration status. Of 120 data points (10 per participant), 43 were in a false positive region (IOP < 13.2 mm Hg, but body mass loss < 2%), 57 were true negatives (IOP > 13.2 mm Hg and body mass loss < 2%), and 11 were true positive (IOP < 13.2 mm Hg and body mass loss > 2%). Overall 57% of the data were correctly classified by these limits, resulting in a test sensitivity of 55% and specificity of 57%.

### ΔIOP

**Figure 2:**
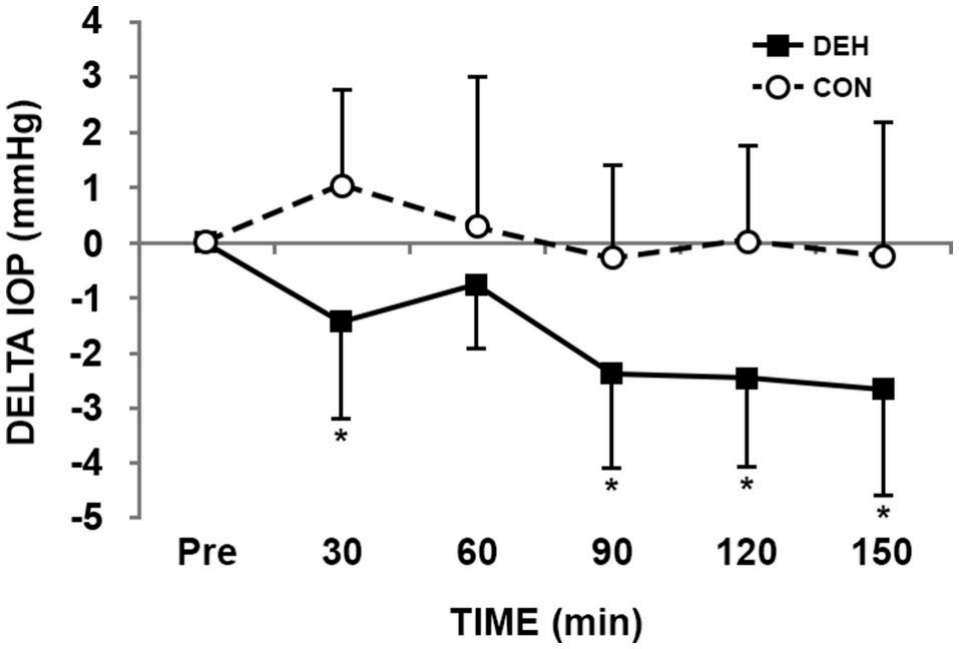
**ΔIOP from baseline in the fluid restriction (DEH) and provision (CON) trials**. Significantly different to control at same time point ^*^p < 0.05.

Normalising the IOP to individual baseline values, ΔIOP (Figure 2), produced significant main effects of trial (CON 0.14 ± 1.9, DEH −1.63 ± 0.77 mm Hg, *p* = 0.002), time (*p* < 0.001) and their interaction (*p* = 0.020). Significant post-hoc comparisons, adjusted for multiple comparisons, were observed at 30, 90, 120, and 150 min (Figure 2).

ΔIOP was significantly related to body mass loss (*r* = 0.526), serum osmolality (*r* = −0.385) and core temperature (*r* = −0.314). Univariate general linear model revealed a significant association for ΔIOP with body mass loss, serum osmolality and core temperature (Table 2). At a 2% loss in body mass, ΔIOP was predicted to be −2.4 mm Hg. Utilizing this cut-off 19 data points were classified as false positives (ΔIOP < −2.4 mm Hg and body mass loss < 2%) and 9 false negatives (ΔIOP < −2.4 mm Hg and body mass loss > 2%). Eleven true positives and 81 true negatives were identified. Using ΔIOP 77% of the data was correctly classified by these limits (sensitivity: 55%; specificity: 81%; Figure 1B).

## Discussion

This study is the first to experimentally evaluate the efficacy and sensitivity of using IOP to assess hydration status following intermittent exercise in the heat, with and without fluid restriction. Assessing thermal hypohydration using ocular fluids has recently gained interest in sports medicine literature (Fortes et al., 2011; Hunt et al., 2012; Sollanek et al., 2012; Sherwin et al., 2015) and IOP, in particular, may be appealing to sports medicine practitioners, clinicians, and researchers because the procedure is non-invasive, causes minimal discomfort, requires minimal training to perform accurately, and provides a reading within seconds. The novel findings of this investigation were: (1) in partial agreement with our initial hypothesis, a statistically significant interaction was observed between IOP and the level of hypohydration; however, there was no difference in IOP at any time during exercise in the heat irrespective of fluid provision or restriction (Table 1), and (2) using an IOP value of 13.2 mm Hg as a criterion reference to assess a 2% loss in body mass resulted in only 57% of the data being correctly classified (Figure 1A). Thus, evidence from the present study does not support the use of IOP as an acute single assessment index of hypohydration.

In accordance with the experimental design, there was a systematic and significantly greater decline in body mass observed in the DEH compared to the CON trial (Table 1), averaging 0.5% per 30 min of treadmill walking. In conjunction with the body mass loss, serum osmolality also increased with progressive dehydration (Table 1) to values associated with a significant hypertonic-hypovolemia (Cheuvront et al., 2010). Hypohydration increases the heat strain experienced by those undertaking physical activity in the heat (Armstrong et al., 1997; Sawka et al., 2001), and previous studies that have induced body mass losses >2% also routinely observed decrements in endurance physical performance (Sawka et al., 2007; Cheuvront and Kenefick, 2014). Therefore, the level of hypohydration observed in the fluid restriction trial of this study was of practical significance.

Fluctuations in IOP result from alterations in the rate of formation of the aqueous humor within the posterior chamber and/or the drainage of the aqueous humor from the anterior chamber of the eye. The rate of aqueous humor drainage is primarily influenced by anatomical structures and venous pressure (Brubaker, 1991) and has been reported to be uninfluenced by exercise (Stewart et al., 1970; Hong et al., 2014). Active transport, ultrafiltration, and diffusion are responsible for the formation of the aqueous humor (Brubaker, 1991). Of these diffusion is thought to be most important during fluid ingestion and/or exercise, as active transport and ultrafiltration have been shown to be uninvolved in acute changes of IOP in these situations (Brucculeri et al., 1999). Water is the main constituent of aqueous humor and it enters the posterior chamber by osmosis (Brubaker, 1991). Hyperosmotic agents (i.e., mannitol, glycerol, and isosorbide) have been shown to reduce IOP by creating a blood-ocular osmotic pressure gradient, thereby lowering the ocular tension via dehydration (Smith and Drance, 1962). Exercise-induced hypohydration also raises plasma osmolality, creating an osmotic gradient, favoring the movement of water from the aqueous humor to the blood. This would reduce the rate of aqueous humor formation and lower IOP (Ashkenazi et al., 1992; Risner et al., 2009). The current study provides empirical evidence to support this mechanism as a statistically significant relationship was found between serum osmolality and ΔIOP (Table 2). The slope of the relationship was negative, indicating that IOP is reduced when serum osmolality is increased. Body mass loss was also significantly associated with both absolute IOP and ΔIOP (Table 2), further supporting the effects of hydration status. Although the CON trial isolated the effects of body water deficit by replicating the absolute exercise intensity, changes in body posture and diurnal effects, it should be noted that a causal relationship cannot be concluded from the associations observed in the current study.

Fluid ingestion has also been shown to influence IOP (Brucculeri et al., 1999; Read and Collins, 2010). Acute ingestion of 1 L of fluid has been documented to cause a 1–2 mm Hg increase in IOP that peaks after 10–15 min and is still elevated at 30 min (Brucculeri et al., 1999; Read and Collins, 2010), but has returned to baseline at a time point between 30 and 45 min (Brucculeri et al., 1999). The increased IOP was postulated to be in response to gastric distension eliciting a sympathetic reflex increase in systemic arterial and vena caval pressure (Brucculeri et al., 1999). The increased vena caval pressure in turn would elevate episcleral venous pressure, minimizing aqueous drainage and subsequently elevating IOP. It is unlikely that the ingestion of water, independent of its influence on hydration status, influenced IOP in the current study as all measurements were recorded > 30 min after the fluid was consumed and the total volume of fluid consumed (376 ± 73 mL) would have produced a significantly smaller degree of gastric distension. Further, given fluid ingestion, irrespective of absorption *per se*, can alter the fluid regulatory response (Figaro and Mack, 1997), additional research is warranted to examine the effect of using a dehydration model that also includes some fluid consumption.

IOP is also known to be reduced following exercise (Risner et al., 2009; Hong et al., 2014). The decline in IOP following short duration high intensity dynamic exercise coincides with the rise in blood lactate and plasma osmolality (Marcus et al., 1970; Stewart et al., 1970). In comparison, it has previously been demonstrated that short duration low intensity exercise produces a small decline in IOP, without these changes in blood lactate and plasma osmolality (Harris et al., 1994). These findings suggest an independent effect of exercise intensity. While blood lactate was significantly correlated with absolute IOP (*r* = 0.190), this relationship became insignificant when corrected for repeated measurements within each participant (Table 2). Similarly, there was no difference in blood lactate between the DEH and CON trials (Table 1). The absolute workload, of 5 km·h^−1^ and 1% grade represented a relative intensity for each participant of 20 ± 6% $\dot{\text{V}}$O_2_ max which was significantly lower than the previous study (Harris et al., 1994) that reported changes in IOP without differences in blood lactate or pH. The absolute workload was also consistent between trials, yet we observed a significant difference in the IOP response to exercise-induced hypohydration (Figure 2). Therefore, it could be postulated that the IOP response occurred independently of aerobic exercise intensity, blood lactate or water consumption, supporting our primary hypothesis that IOP is reduced to a greater extent during exercise in the heat with fluid restriction, concomitant with modest hypohydration (2–3% body mass loss) and increased serum osmolality.

Some thermoregulatory and cardiovascular variables differed between the DEH and CON trial and should be considered as potential factors influencing the IOP response. The present study observed a significantly elevated core temperature in the DEH trial compared to the CON trial from the 90 min time period to the end of the trial. The magnitude of this effect was on average 0.3°C, range 0.1–0.8°C (Table 1). This elevation is a normal thermoregulatory response to exercise in the heat with fluid restriction; however, it does indicate a potential confounder to the above conclusion. It could be argued that the IOP response observed may be influenced by core temperature instead of hydration status *per se*, with a negative correlation observed with ΔIOP (*r* = −0.314, *p* < 0.001) but not between absolute IOP and core temperature (*r* = −0.075, *p* = 0.383) (Table 2). Heart rate was also increased from 120 min in the DEH trials compared to CON (Table 1). However, there was no significant relationship between absolute (*r* = −0.003, *p* = 0.976) or ΔIOP (*r* = −0.143, *p* = 0.119) with heart rate. Our findings are supported by other researchers who have previously observed no relationship between heart rate and IOP (Ashkenazi et al., 1992; Karabatakis et al., 2004), but a negative association been ΔIOP and core temperature (Hunt et al., 2012).

Although the current data suggest an association between IOP and hydration status, there is limited potential for IOP to be used as a simple and practical technique to indicate hydration status in non-clinical settings (i.e., sporting or occupational environments). A body mass loss of 2% was chosen as a criterion level of hypohydration, as this level has previously been associated with decrements in physical endurance performance, increased heat strain, and increased risk of developing heat illness (Armstrong et al., 1997; Sawka et al., 2001; Cheuvront and Kenefick, 2014). Using the relationship between body mass loss and IOP, the corresponding IOP cut-off was predicted to be 13.2 mm Hg. The application of these cut-off limits to the IOP and body mass loss relationship can be observed in Figure 1A and highlight only 57% of the data was correctly classified with these limits. IOP at baseline ranged between 8.5 and 22 mm Hg, while in agreement with population norms (David et al., 1992) this does highlight a large degree of inter-individual variability. Three participants (25%) had an IOP lower than the cut-off when adequately hydrated at baseline. Further as the trial progressed, all participants evidenced a decrease in IOP, however, the IOP of four participants (33%) did not fall below the cut-off limit in spite of becoming dehydrated (evidenced by > 2.5% body mass loss). This suggests that the individual variability in IOP may be too large to establish a set limit value to indicate hypohydration without a euhydrated criterion baseline. Further, in comparison to other commonly used markers to diagnose exercise-induced hypohydration of ≥ 2% body mass loss (Muñoz et al., 2013), serum (sensitivity: 83%, specificity: 82%), saliva (86, 91%) and urine (83, 83%) osmolality, and urine volume (79, 79%) and specific gravity (81, 81%) all have been shown to have greater sensitivity and specificity compared to the IOP results presented within this study (55, 57%).

Despite the high individual variability in IOP a decline during the exercise-induced hypohydration was observed in all the participants. Therefore, we examined the use of a change score, from baseline, as a potential indicator of a change in hydration status. Using the relationship between body mass loss and ΔIOP from baseline, a 2% body mass loss corresponded to ΔIOP of −2.4 mm Hg and slightly improved the classification accuracy to 77% (Figure 1B) and the test specificity (81%), but not the sensitivity (55%). The limited number of observations >2% body mass loss (16% of the data) in the current study significantly influences the IOP test sensitivity, regardless its diagnostic ability in the current study was only slightly better than random chance.

In conclusion, IOP is progressively reduced during exercise-induced hypohydration, but remains stable if hydration is maintained during exercise in the heat. The present study provides novel evidence to suggest that IOP is significantly correlated to hydration status, likely due to the effect of a rise in serum osmolality on the rate of formation of aqueous humor. However, large inter-individual variability in baseline IOP and in the IOP response to progressive dehydration prevent IOP use, as measured by rebound tonometry, as an acute single assessment marker of hydration status.

## Author contributions

IS and JC conceived and designed the research, analyzed the data and drafted the manuscript. BD, DB, and AB performed the experiments. IS, JC, and BF interpreted results of the experiments. All authors edited and revised the manuscript. All authors approved the final version of the manuscript and agree to be accountable for all aspects of the work in ensuring that questions related to the accuracy or integrity of any part of the work are appropriately investigated and resolved. All persons designated as authors qualify for authorship, and all those who qualify for authorship are listed.

### Conflict of interest statement

The authors declare that the research was conducted in the absence of any commercial or financial relationships that could be construed as a potential conflict of interest.
